# Influence of social support on cognitive change and mortality in old age: results from the prospective multicentre cohort study AgeCoDe

**DOI:** 10.1186/1471-2318-12-9

**Published:** 2012-03-20

**Authors:** Marion Eisele, Thomas Zimmermann, Mirjam Köhler, Birgitt Wiese, Kathrin Heser, Franziska Tebarth, Dagmar Weeg, Julia Olbrich, Michael Pentzek, Angela Fuchs, Siegfried Weyerer, Jochen Werle, Hanna Leicht, Hans-Helmut König, Melanie Luppa, Steffi Riedel-Heller, Wolfgang Maier, Martin Scherer

**Affiliations:** 1 Department of Primary Medical Care, Center for Psychosocial Medicine, University Medical Center Hamburg-Eppendorf, Martinistraße 52, 20246 Hamburg, Germany; 2 Institute for Biometrics, Hannover Medical School, Carl-Neuberg-Str. 1, 30623 Hannover, Germany; 3 Department of Psychiatry, University of Bonn, Sigmund-Freud-Straße 25, 53105 Bonn, Germany; 4 Department of Psychiatry, Technical University of Munich, 81675 Munich, Germany; 5 Department of General Practice, Medical Faculty, University of Dusseldorf, 40225 Dusseldorf, Germany; 6 Central Institute of Mental Health, Postfach 122120, 68072 Mannheim, Germany; 7 Department of Medical Sociology and Health Economics, University Medical Center Hamburg-Eppendorf, Martinistraße 52, 20146 Hamburg, Germany; 8 Institute of Social Medicine, Occupational Health and Public Health, University of Leipzig, Philipp-Rosenthal-Straße 55, 04103 Leipzig, Germany; 9 DZNE, German Center for Neurodegenerative Diseases, Holbeinstraße 13-15, 53175 Bonn, Germany

## Abstract

**Background:**

Social support has been suggested to positively influence cognition and mortality in old age. However, this suggestion has been questioned due to inconsistent operationalisations of social support among studies and the small number of longitudinal studies available. This study aims to investigate the influence of perceived social support, understood as the emotional component of social support, on cognition and mortality in old age as part of a prospective longitudinal multicentre study in Germany.

**Methods:**

A national subsample of 2,367 primary care patients was assessed twice over an observation period of 18 months regarding the influence of social support on cognitive function and mortality. Perceived social support was assessed using the 14-item version of the FSozU, which is a standardised and validated questionnaire of social support. Cognition was tested by the neuropsychological test battery of the Structured Interview for the Diagnosis of Dementia (SIDAM). The influence of perceived support on cognitive change was analysed by multivariate ANCOVA; mortality was analysed by multivariate logistic and cox regression.

**Results:**

Sample cognitive change (N = 1,869): Mean age was 82.4 years (SD 3.3) at the beginning of the observation period, 65.9% were female, mean cognition was 49 (SD 4.4) in the SIDAM. Over the observation period cognitive function declined in 47.2% by a mean of 3.4 points. Sample mortality (N = 2,367): Mean age was 82.5 years (SD 3.4), 65.7% were female and 185 patients died during the observation period. Perceived social support showed no longitudinal association with cognitive change (F = 2.235; p = 0.135) and mortality (p = 0.332; CI 0.829-1.743).

**Conclusions:**

Perceived social support did not influence cognition and mortality over an 18 months observation period. However, previous studies using different operationalisations of social support and longer observation periods indicate that such an influence may exist. This influence is rather small and the result of complex interaction mechanisms between different components of social support; the emotional component seems to have no or only a limited effect. Further research is needed to describe the complex interactions between components of social support. Longer observation periods are necessary and standardised operationalisations of social support should be applied.

## Background

Social support is known to have a beneficial effect on physical and mental health in old age [1-3]. Several studies revealed an association between a lack of social network and mortality [4,5]. Results regarding the influence of social support on cognition in old age are less consistent. The reason for this is that definitions of social support vary considerably across studies and that operationalisations are not standardised: while some definitions consider structural aspects of social networks such as size [6], or focus on emotional components such as the availability of a good friend with whom to talk [7], others include both social network and emotional components [8-12].

Understood as the number of social relationships of an individual, social support has been found to influence cognitive change and dementia in old age. Several studies show that individuals with less relationships have a higher risk for cognitive decline and dementia than those with more social relations [8,10,11], and that the risk for cognitive decline decreases with an increase in the number of personal contacts [8,9]. Even though evidence for an influence prevails, Seeman and colleagues found no influence of the number of relationships on cognition [12]. However, the operationalisation of structural aspects of social networks is limited since the number of persons a study participant has contact with is not necessarily related to the amount of social support he perceives.

Studies focusing on emotional aspects of social support instead of, or in addition to, structural aspects of social networks are less consistent in their findings. Zunzunegui and colleagues and Béland and colleagues investigated the influence of membership of a social group on cognition. They found that persons who belonged to any kind of social group showed less cognitive decline in old age. This effect increases with age [8,13]. Findings regarding emotional support, understood as having the feeling that there is someone to rely on if needed, are most inconsistent. While cross-sectional studies revealed an association between the presence of a "significant other" and cognition [7,14], only one of three longitudinal studies [9,10,12] reported a positive influence of the perceived sufficiency of support received on cognition [12].

In summary, the influence of social support on cognition remains unclear. Evidence is strongest for a positive influence of large personal social networks and a high frequency of contacts with persons in these networks. However, studies regarding the influence of emotional components of social support on cognition vary in study design, outcomes and operationalisations employed. Especially longitudinal studies vary considerably in their results. Even among studies with comparable outcomes it still remains unclear which particular aspects of social support influence cognition: Does social support impact on cognition through emotional benefits such as stress reduction, or rather through an increased level of physical and cognitive activation due to a large number of friends?

The aim of this study was to investigate the longitudinal impact of the emotional component of social support on cognitive change. We defined emotional support as subjectively perceived support, which is independent from actually received support and structural aspects of social networks and as such excludes influences of increased physical and cognitive activation on cognition. Perceived social support was operationalised using a standardised instrument. Mortality was investigated as a second endpoint, since severe cognitive decline may result in dementia and finally death.

## Methods

### Design

This study is part of the German prospective longitudinal multicentre study on Ageing, Cognition and Dementia in Primary Care Patients (AgeCoDe), established to investigate risk factors for dementia. The AgeCoDe cohort comprises of primary care patients aged 75 years and older. Trained interviewers (psychologists and physicians) visited the patients at home for baseline assessment as well as three follow-up assessments every 18 months. General practitioners were asked to fill out a questionnaire about their patients' health status at each time of assessment. Because perceived social support was assessed for the first time in follow-up 2, all data analysed in this study refer to follow-up 2 and 3.

### Ethics

The study was conducted in accordance with the Helsinki declaration and has been approved by the local ethics boards of all participating centres (reference numbers: 050/02 [University of Bonn], 2079 [Faculty of Medicine, University of Düsseldorf], OB/08/02 [Hamburg Medical Association], 143/2002 [Faculty of Medicine, University of Leipzig], 0226.4 [Medical Ethics Commission II, University of Heidelberg at the University Medical Center of Mannheim], 713/02 [Faculty of Medicine, Technical University of Munich]. All participants gave written informed consent prior to study entry.

### Sample

Our analyses are based on a subsample of the AgeCoDe cohort. AgeCoDe study participants were recruited from 138 primary care practices at six German study centres (Hamburg, Bonn, Düsseldorf, Leipzig, Mannheim and Munich) between January 1st 2003 and November 30th 2004. Inclusion criteria were absence of dementia (according to the general practitioner's opinion) and at least one consultation with the general practitioner within the last 12 months. All participants had to be regular patients of the respective primary care practices. Exclusion criteria were residence in a nursing home, consultations by home visit only, severe illness fatal within three months (according to the general practitioner's opinion), insufficient ability to speak German, deafness, blindness and an insufficient ability to consent. A total of 3,327 primary care patients were included in the AgeCoDe cohort and participated in the baseline assessment. Follow-up assessments were conducted at 18 months intervals. This study assesses the data of follow-up 2 (FU2) and follow-up 3 (FU3) which were performed 3 and 4.5 years after baseline assessment, respectively.

#### Sample mortality

**Figure 1:**
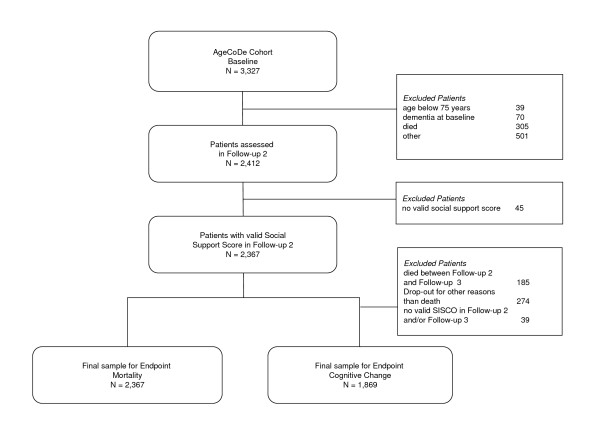
**Sampling frame**.

At FU2, 2,412 of 3,327 patients initially enrolled at baseline were assessed. Patients not assessed at FU2 were excluded for the following reasons: 39 patients were younger than 75 years at baseline, 70 patients had a diagnosis of dementia at baseline, 305 patients died and 501 patients dropped out for other reasons. All patients assessed at FU2 were required to have a valid social support score (no more than 3 out of 14 missing items), which was met by 2,367 patients (out of 2,412 patients; 98.1% [see Figure 1]).

#### Sample cognitive change

To investigate the influence of perceived support on cognitive change, patients were additionally required to survive until FU3 and to have a valid score for cognition at FU2 as well as FU3. This applied to 1,869 patients, which is 77.5% of all patients assessed at FU2 (see Figure 1).

### Operationalisation of social support

This study investigates perceived social support, as the emotional component of social support, operationalised using the 14-item short form of the questionnaire for social support (FSozU K-14) by Fydrich and colleagues [15]. This instrument measures perceived social support independently of actually received support. Examples of the 14 items are: "I have a very close person, on whose help I can always count", "I know several people, with who I enjoy to spend time with", "When needed I have no trouble to borrow things from my neighbours" and "I have friends/family members who take the time and definitely listen to me, when I need to talk".

The 5-item Likert scale was adapted for the assessment of elderly patients with probable cognitive impairment to include yes/no answers, according to the suggestion by Kelsey and colleagues [16]. For analysis, a sum score was calculated ranging from 0-14 with high scores indicating a high level of perceived social support.

The distribution was left-skewed and, therefore, normal distribution could not be taken for granted. For that reason the score was dichotomised. The cut-off point was chosen at the elbow of the distribution at 11.5 points. This allowed expedient differentiation in regard to context and, at the same time, ensured a group size sufficient for statistical analysis (low social support 24.8%, N = 587; high social support 75.2%, N = 1,780).

Because physical and cognitive activation stimulated by social contacts can also influence cognition, we included both factors as potential confounders. Physical activity was defined as performing at least one physical activity at least twice a week (e.g. riding a bicycle, taking longer walks, hiking, swimming, etc.). Cognitive activity was defined as being at least twice a week engaged in at least one of the following activities: reading, writing, solving crossword puzzles and memory training.

### Cognitive function

Cognitive function was assessed by the neuropsychological test battery of the Structured Interview for the Diagnosis of Dementia of the Alzheimer type, Multi-infarct Dementia and Dementia of other Aetiology according to DSM-III-R, DSM-IV and ICD-10 (SIDAM) [17]. The SIDAM Score (SISCO) was calculated from the 55-item neuropsychological test battery. Cognitive change within the observation period of 18 months was measured by calculating the difference between the SISCO at FU3 and at FU2. The Mini Mental Status Test (MMST) is included in SIDAM [18]; scores were calculated accordingly.

### Mortality

If a patient could not be reached by mail and phone to schedule the next assessment, a contact person (usually spouse, children, other relatives or the general practitioner) was phoned. In case of death the contact person was asked to provide the date of death.

### Health status

Data regarding health status were collected through patient interviews and questionnaires for the general practitioners. Subjective health status was measured using the visual analogue scale EQ-VAS of the EQ-5D in its German translation [19]. Cognitive and physical activity and impaired ability to walk were assessed by patient interviews. In addition, patients were asked to provide their current height and weight; the Body Mass Index was calculated based on this information. For each patient, the general practitioner filled out a questionnaire regarding patient morbidity. Based on the questionnaires, objective health status was measured by the number of co-morbidities and relevant chronic diseases in this age group. Additionally, patients were asked to show which medications they take in order to calculate the number of medications taken. Because cardiovascular diseases are a risk factor for mortality, a sum score for cardiovascular illness was included in the analysis. Cardiovascular illness was defined as the number of cardiovascular conditions a patient has. The following cardiovascular conditions were assessed: hypertension, arrhythmia, coronary heart disease, myocardial infarction, peripheral artery occlusive disease, stenosis of precerebral arteries, transient cerebral ischaemic attack and cerebral infarction. Alcohol misuse/abuse was measured by the judgement of the general practitioner.

### Psychosocial factors

Information regarding marital status, engagement in social groups (e.g. local community, church), smoking behaviour and sensory impairment were assessed by patient interviews. The instrumental activities of daily living (IADL) were assessed by the Instrumental Activities of Daily Living Scale by Lawton and Broady [20]. Depression was assessed using the 15-item short form of the Geriatric Depression Scale [21]. Social status was measured in terms of education according to the CASMIN classification [22].

For patients with a MMST score below 25, all information obtained through patient interviews was double-checked for accuracy by a close relative or, if not available, by nursing staff or the general practitioner.

### Statistical analysis

All statistical analyses were performed with SPSS version 16.0. Multifactorial ANCOVA was applied to analyse the influence of social support on cognitive change controlled for confounders. The set of confounding variables regarding cognitive change in old age was selected based on literature research and included the following confounders: age [7,13,14]; gender [23,24]; education [7,25]; cognitive function at FU2 [26]; sensory impairment (Bassuk, Glass, 1999) [10]; health status in terms of number of physical co-morbidities, number of medications taken and self-rated health status [13,14]; physical activity [27-29]; cardiovascular illness and alcohol abuse [30]; depression [31]; diabetes mellitus, smoking behaviour, Body Mass Index [30]; cognitive activity [32,33]; instrumental activities of daily living [7]; engagement in social groups [8,13,14]; as well as interaction effects between age and gender [8] and between age and engagement in social groups [13,14]. Results were double-checked with other sets of confounding variables. Cognitive and physical activity were included in the set of confounding variables in addition to social support, because they are potentially effective components of social support. The research question focuses on the influence of perceived social support on cognitive change. The examination of the confounding factors was not the objective. Therefore, superfluous variables without significant influence were excluded from the model by backward selection.

Multifactorial cox and logistic regression models were calculated to analyse the impact of perceived social support on mortality and survival time controlled for confounding variables. The set of confounding variables was selected based on literature research. Confounding variables influencing cognition in old age were included as well, because severe cognitive decline may result in dementia and reduce survival time. The set contained the following confounders in addition to the set of confounding variables for cognitive change: marital status [10,34]; social stratum (by education) [35]; as well as interaction effects between gender and marital status [34] and between engagement in social groups and gender [8]. Physical and cognitive activity were included for a detailed investigation of potentially effective components of social support. The final model was selected by forward as well as backward selection and was double-checked with other sets of variables.

## Results

### Cognitive change

Of 1,869 patients eligible for analysis regarding cognitive change, 65.9% were female and 34.1% male. The mean age at FU2 was 82.4 years (SD 3.3, range 79-95). This subsample was smaller than the sample analysed regarding mortality since all patients who died or dropped out for any other reasons between FU2 and FU3 were excluded (see Figure 1). Mean cognitive function was 49 (SD 4.4) out of 55 possible points in the SISCO. In total, cognition declined in 47.2% of the patients by a mean of -3.4 (SD 3.4) points in the SISCO and cognition improved in 38.2% of the patients by a mean of 2.3 (SD 1.6) points.

Patients assessed at FU2 but not included in the sample for cognitive change were somewhat older (mean age 83.2 years (SD 3.8), T = 4.617; p = 0.000), more likely to be female (66.1%, *χ*^2 ^= 0.007; df = 1; p = 0.932) and had worse cognitive function at FU2 (46.0; SD 7.9) points in the SISCO, T = -8.310; p = 0.000) than included patients.

Of all patients eligible for analysis, 24% had low and 76% had high perceived social support. Cross sectional analyses revealed an association between perceived support and cognitive function at FU2 (T = -2,564; p = 0.011). The longitudinal influence of social support was analysed by investigating cognitive change between FU2 and FU3.

**Figure 2:**
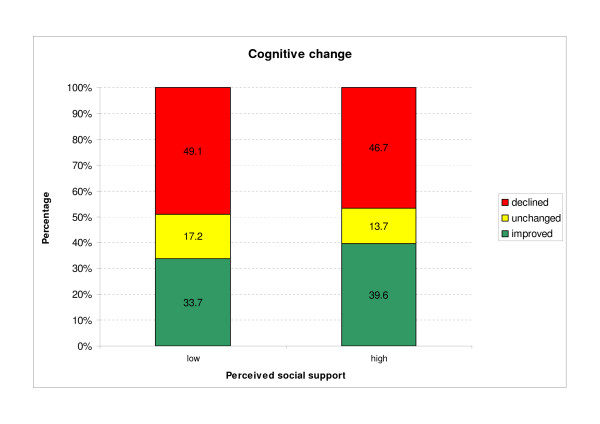
**Cognitive change subject to perceived social support**.

Figure 2 shows cognitive change in relation to high and low social support. Both groups differ regarding the percentage of patients whose cognitive function declined, stayed unchanged, or improved (*χ*^2 ^= 6.361; df = 2; p = 0.042). In both groups, cognitive function slightly declined between FU2 and FU3 (high social support -0.6; SD = 3.5 and low social support -1.0; SD = 4.2). The decline was significantly higher in the group with low social support (T = -2.058; p = 0.048).

The final model of the multivariate ANCOVA (Model 1) is displayed in Table 1. To determine whether the inclusion of physical and cognitive activity as possible influencing factors of social support changes the influence of perceived support in the final model, the final model was additionally calculated including both factors (see Model 2 in Table 1). In both models, perceived social support did not have a significant influence on cognitive change over the observation period of 18 months (Model 1: p = 0.135; Model 2: p = 0.146). Cognitive change was significantly influenced by gender, age, impaired ability to walk, instrumental activities of daily living, cognitive function at the beginning of the observation period and Body Mass Index. Physical and cognitive activity had no significant influence.

**Table 1:** Final models for the endpoint cognitive change (ANCOVA, backward selection)

	Model 1		Model 2	
Variable	F-ratio	Significance	F-ratio	Significance
Constant	0.125	p = 0.724	0.159	p = 0.690
Perceived social support	2.235	p = 0.135	2.114	p = 0.146
Gender	29.596	p = 0.000	29.584	p = 0.000
Age	5.747	p = 0.017	5.746	p = 0.017
Impaired ability to walk	3.398	p = 0.017	3.102	p = 0.026
Instrumental activities of daily living	51.832	p = 0.000	51.973	p = 0.000
Cognitive function at FU2	5.841	p = 0.016	5.497	p = 0.019
Body Mass Index	13.523	p = 0.000	13.796	p = 0.000
Cognitive inactivity			1.013	p = 0.314
Physical inactivity			0.516	p = 0.473

Dependent Variable: Cognitive Change (FU3-FU2)

### Mortality and survival time

Of 2,367 patients eligible for analysis regarding mortality, 65.7% were female and 34.3% male. The mean age at FU2 was 82.5 years (SD 3.4, range 77-101 years). Mean cognitive function was 48.6 (SD 4.8) out of 55 points in the SISCO.

**Figure 3:**
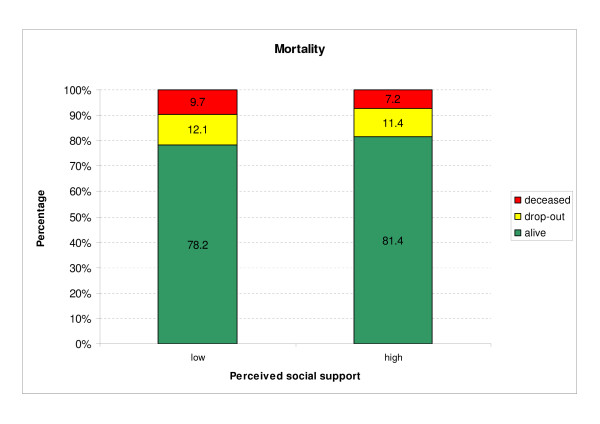
**Mortality between FU2 and FU3 subject to perceived social support**.

Of all patients, 24.8% had low and 75.2% had high perceived social support. During the 18 months observation period, 185 patients (7.8%) died and N = 1,908 (80.6%) survived. A total of N = 274 patients (11.6%) dropped out between FU2 and FU3 for other reasons than death. Figure 3 shows the percentage of mortality in patients with high and low perceived social support. The percentage of survivors is significantly higher in the group with high perceived social support (*χ*^2 ^= 3,899; df = 1; p = 0.049).

Patients excluded at FU2 for the lack of a valid social support score were mostly female (82.2%, *χ*^2 ^= 5.400; df = 1; p = 0.020), were slightly older (mean age 84.2; SD 4.4) years, T = 2.587; p = 0.013) and performed worse in cognitive testing (32.5; SD 15.6) points in the SISCO, T = -5.906; p = 0.000).

Table 2 and Table 3 show the final models for factors influencing mortality and survival time. Perceived social support does not significantly influence mortality (p = 0.332, Table 2 Model 3) and survival time (p = 0.216, Table 3 Model 5). Factors with a significant influence on mortality were higher age, lower cognitive function at the beginning of the observation period, lower subjective health status, alcohol abuse, little physical activity and impaired instrumental activities of daily living. The same factors were found to significantly influence survival time (see Table 3). Regular cognitive activity had no significant influence on mortality (see Table 2 Model 4) and survival time (see Table 3 Model 6). The same factors were found to significantly influence mortality and survival time if selected by backward selection.

**Table 2:** Final models for the endpoint mortality (logistic regression, forward selection)

	Model 3			Model 4		
Variable	Odds Ratio	Significance	95% Confidence interval	Odds Ratio	Significance	95% Confidence interval
Constant	0.002	p = 0.006		0.003	p = 0.008	
Perceived social support	1.202	p = 0.332	0.829 - 1.743	1.212	p = 0.312	0.835 - 1.757
Age	1.109	p = 0.000	1.059 - 1.161	1.106	p = 0.000	1.056 - 1.158
Cognitive function at FU2	0.968	p = 0.038	0.939 - 0.998	0.966	p = 0.030	0.937 - 0.997
Health status (subjective)	0.987	p = 0.004	0.978 - 0.996	0.987	p = 0.006	0.978 - 0.996
No alcohol abuse	0.252	p = 0.010	0.088 - 0.715	0.244	p = 0.008	0.086 - 0.695
Physical inactivity	2.183	p = 0.000	1.548 - 3.079	2.226	p = 0.000	1.580 - 3.138
Preserved instrumental activities of daily living	0.790	p = 0.000	0.722 - 0.864	0.790	p = 0.000	0.723 - 0.864
Cognitive inactivity				0.666	p = 0.362	0.278 - 1.597

Dependent Variable: Mortality between FU2 and FU3

**Table 3:** Final models for the endpoint survival time (cox regression, forward selection)

	Model 5			Model 6		
Variable	Exp(B)	Significance	95% Confidence interval	Exp(B)	Significance	95% Confidence interval
Perceived social support	1.241	p = 0.216	0.882 - 1.748	1.240	p = 0.217	0.881 - 1.745
Age	1.099	p = 0.000	1.054 - 1.146	1.098	p = 0.000	1.054 - 1.145
Cognitive function at FU2	0.974	p = 0.040	0.950 - 0.999	0.970	p = 0.022	0.944 - 0.995
Health status (subjective)	0.986	p = 0.002	0.977 - 0.995	0.987	p = 0.003	0.978 - 0.995
No alcohol abuse	0.274	p = 0.005	0.111 - 0.674	0.268	p = 0.004	0.109 - 0.660
Physical inactivity	2.149	p = 0.000	1.557 - 2.967	2.187	p = 0.000	1.585 - 3.018
Preserved instrumental activities of daily living	0.809	p = 0.000	0.746 - 0.876	0.808	p = 0.000	0.746 - 0.875
Cognitive inactivity				0.619	p = 0.237	0.279 - 1.372

Dependent Variable: Survival time between FU2 and FU3

## Discussion

### Main results

The aim of this study was to investigate the longitudinal impact of perceived social support on cognitive change and mortality in old age. Perceived social support, understood as the emotional component of social support, was not found to significantly influence cognitive change, mortality and survival time over the 18 months observation period.

So far, the mechanisms of how social support may act as a protective factor remain unclear. This is mainly due to inconsistent operationalisations applied among studies reporting an association between social support and cognition. Three mechanisms are conceivable to account for the effect of social support on cognition: 1. physical activation through living a socially active lifestyle (e.g. leaving the house more often to meet friends), 2. cognitive stimulation through social interaction, and 3. positive emotions caused by perceived social support, which may decrease stress levels.

This study focused on three aspects of social support in order to investigate which of these components, if any, influence cognitive change: perceived social support as the emotional component of social support, and physical as well as cognitive activity as factors that possibly act through activation. Our results indicate that none of the three components had a significant influence on cognitive change over the 18 months observation period. Only physical activity was found to influence mortality and survival time.

### Strengths and limitations

This study was performed on a large subsample of the AgeCoDe cohort. The mean age of participants was 82 years. A large number of variables was available to control the influence of social support on cognitive change and mortality for confounders. Particular strengths of this study are the high quality of data and the high level of quality assurance. Data were collected in face-to-face patient interviews. All information provided by patients with impaired cognitive function (MMST < 25) was double-checked for accuracy by their spouse, relatives, nursing staff or the general practitioner. If a patient could not be reached by mail and phone to schedule the next assessment, a contact person (usually spouse, children, other relatives or the general practitioner) was phoned. In case of death the contact person was asked to provide the date of death. Social support was assessed by a validated instrument, the FSozU-K14 by Fydrich and colleagues [15], in patient interviews. The mean cognitive function of patients was 48.6 (SD 4.8) out of 55 points in the SISCO at the time of assessment of perceived social support. Since the cut-off value for early-stage dementia in the assessed age group is 36 points [36], we assume that patients were able to provide accurate answers to the social support items.

The assessed sample is unique in presenting data regarding the influence of social support on cognition and mortality for the 80+ age group. However, at the same time, the mean age of 82 years of participants limits comparisons to other studies focusing on younger age groups. It has been suggested that the influence of social support changes with increasing age. Béland and colleagues report that a cross-sectional association between social support and cognition exists until the age of 80 years but disappears beyond the age of 80 [13]. This effect needs further investigation. Patients with significantly worse cognition had to be excluded from analyses because no social support score was available. However, all models were controlled for cognitive performance at the beginning of the observation period and, therefore, influence of perceived support was measured independently from this.

Further limitations of this study are the observation period and the mortality rate among the 80+ years age group. Compared to other studies, the 18 months observation period is rather short. As a result, small effects of social support on cognition may not have been detectable. In our subsample, the death rate of 7.8% is relatively low compared to the drop-out rate of 11.6%. We therefore considered survival time in addition to mortality itself to make results more reliable.

Co-morbidity was assessed by the number of existing co-morbidities and the number of medications taken per patient, rather than by specific illnesses. In addition, we included the subjective health status in the statistical model. Subjective health status has been shown to be a valuable predictor of mortality, with similar predictive power to co-morbidity scores [37].

One last limitation of this study is the skewed distribution of the social support score. The score did not adequately differentiate among patients with a high social support score and, as a result, we dichotomised the score. However, we additionally performed all analyses with the original social support score (not displayed). The results were consistent with the findings presented in this article.

### Comparison with literature

#### Cognitive change

We identified a cross-sectional and a longitudinal univariate association between perceived social support and cognitive change. This association could not be found in the multifactorial longitudinal model. Longitudinally, perceived social support was influenced by age, gender, impaired ability to walk, instrumental activities of daily living, Body Mass Index and cognitive function at the beginning of the observation period. These findings are consistent with previous studies [7,10,13,24,26,30].

Seeman and colleagues [12] used a definition of social support similar to our own ("How often do you feel loved by family/friends? How often are family/friends/your partner ready to listen if you need to speak about problems?"). They found a significant influence of emotional support on cognitive change over an observation period of 7.5 years among patients aged 70-79 years (mean age 74 years at baseline). However, the cognitive change measured was deemed rather small by the authors. The results of the linear regression showed no significant predictive value of emotional support (p = 0.07) when controlled for further social network and support characteristics as well as sociodemographic variables. Only after reducing the model by eliminating all other social components, emotional support yielded a significant p-value (p = 0.05, b = 1.2) and an explained variance of 0.3%. In total, evidence for the impact of emotional support on cognitive change in this study is weak. In view of both the explained variance of 0.3% and the significantly longer observation period (compared to our study), it does not surprise that we did not find a significant influence of emotional support in our study. A second longitudinal study by Bassuk et al. investigated the influence of the emotional component of social support over 12 years among a younger sample (65+ years) [10]. The emotional component of social support had no significant influence on cognitive change. By contrast, when controlled for adequacy of emotional support, lack of social integration did have a significant negative influence. The authors concluded that the influence of social network size does not act via the emotional component of social support. This conclusion is consistent with our results. A third longitudinal study by Green and colleagues revealed a negative effect of emotional support and frequency of social contacts on cognitive change [23]. The authors concluded that social support does not have an influence on cognitive change, but that cognitive change influences social support. This association could not be confirmed by our study. Possible reasons are the significantly longer observation period (10 years vs. 18 months in our study) and the considerably lower mean age of study participants (47 years vs. 82.5 years in our study).

In summary, our study did not reveal a positive influence of perceived social support (as the emotional component of social support) on cognitive change in old age. This may be due to the short observation period of 18 months, or else it may not be the emotional component but other aspects of social support that influence cognition.

#### Mortality

Univariate models showed an association between perceived social support and mortality as well as survival time. In multifactorial models this association was no longer significant. Longitudinally, mortality and survival time were both influenced by higher age, lower cognitive function at the beginning of the observation period, lower subjective health status, alcohol abuse, little physical activity and impaired instrumental activities of daily living. These results confirm findings from previous studies [35,38-41].

A longitudinal study by Rodriguez-Laso and colleagues [42] investigated the influence of emotional support on mortality over an observation period of 6 years. The mean age of the sample was 71 years. Emotional support was defined as the feeling of being loved/accepted and the feeling that other people listen if there is a need to talk about something. They did not find a significant influence of emotional support on cognition. The presence of a "significant other" reduced the mortality risk by 25%. Marriage did not significantly influence mortality. In contrast, Baumann found marriage to be a protective factor in men in a study among patients aged 55-75 years over an observation period of 5 years [34]. In our study, neither perceived support nor married status had a significant influence on mortality and survival time, which may be due to the higher mean age of our sample and the shorter observation period.

### Possible mechanisms

Fratiglioni and colleagues discuss three hypotheses of how social support may protect against cognitive decline: (1) The cognitive reserve hypothesis, (2) the stress hypothesis and (3) the vascular hypothesis [43]. (1) The cognitive reserve hypothesis states that social activity leads to increased mental stimulation of the brain and increased synaptogenesis in adulthood. In damaged areas of the brain (e.g. Alzheimer's Pathology) either cells are able to work more efficiently, or surrounding areas take over functions of the affected areas. This hypothesis is supported by the finding of Bennett and colleagues that the size of a social network has a mediating effect on the clinical symptoms of existing Alzheimer's pathology [6]. Our results indicate that complex cognitive activity in a social context may be necessary for these positive effects, while mental activity independent of a complex social environment may be less effective. Different to previous studies we examined the outcome of cognitive change rather than dementia. The positive effects of preceding mental stimulation may only be detectable in established Alzheimer's pathology but not in pre-pathological stages. Brain damage in our patients may not have been severe enough to measure the positive effects of mental stimulation.

(2) The stress hypothesis focuses on the emotional component of social support. Perceived social support is assumed to lead to a positive self-image and better self-esteem. This may buffer stress in anxiety provoking situations which have been related to Alzheimer's disease [44]. An increased glucocorticoid production, as observed in maladaptive reactions to stress, has been shown to cause hippocampal damage which leads to impaired learning and memory function. This hypothesis could explain the protective effect of perceived support for cognitive decline independent of increased levels of social activation. Our results do not support this hypothesis. However, as stated above, in patients showing cognitive decline during our observation period, brain damage may not have been severe enough to detect a possible effect.

(3) The third hypothesis assumes that large social networks stimulate physical activity, which decreases the risk of cardiovascular events. Vascular diseases as well as vascular risk factors are involved in the pathogenesis and progression of Alzheimer's dementia [45,46]. This hypothesis could explain why the emotional component of social support may be ineffective if investigated independently of social activity, while social network size, as a proxy for activity levels, has a positive effect. We found neither the emotional component of social support, nor physical activity to have an influence on cognitive change over 18 months, but we did identify an influence of physical activity on mortality. A longer observation period and more marked differences in cognitive change may be necessary to investigate this hypothesis further.

### Implications for practice

For practice, the findings of this study implicate that perceived social support as a stand-alone factor is insufficient to protect against cognitive decline and mortality in old age. A protective effect may be true for physical and cognitive activity in social contexts. However, this study only focused the influence of social support on cognitive change and mortality. We did not assess the potentially important impact of social support on health status, quality of life and depression in old age patients. Loneliness, understood as a lack of social support, is known to be one of the major challenges in the oldest old and should be addressed in practice. Therefore, social interaction and social integration still should be encouraged in old age.

### Implications for future research

In sum, the results of previous studies are inconsistent. While some studies found a protective effect of social support on cognitive change and mortality, others did not. In the present study, multivariate models did not reveal a protective effect of perceived social support, although a significant effect was found in bivariate analysis. Physical and cognitive activity as potentially effective components of social support could not be shown to have a protective effect on cognitive change, either. Only physical activity had a significant influence on mortality in the multifactorial models, but this effect seems to be independent of the presence or absence of perceived social support. The possible mechanisms of how social support acts on a biological level discussed all refer to dementia. As a result, the cognitive changes investigated in this study may not have been severe enough to allow final conclusions regarding the discussed hypotheses. An influence of social support on cognitive change and mortality through the different components: emotional support, cognitive and physical activity, cannot be explained independently of social context in this sample of patients aged 80 years and over. If existent, the influence of social support on cognition and mortality seems to be more complex.

Future research should address the different components of social support (physical and cognitive activity should be assessed within a social context) as well as more complex models of social support. The effective components of social support need to be identified before well-directed intervention studies are planned. Patients over the age of 80 years should be given special attention in future studies. Previous research indicates that the effect of the emotional component of social support on cognition is rather small. To investigate this effect further, longer observation periods are needed.

## Conclusion

This study did not find a positive influence of perceived social support on cognition and mortality among patients aged 80 years and older.

Previous studies using different operationalisations of social support suggest that an influence of social support on cognitive change may exist. However, the effect may be rather small and is most likely caused by complex mechanisms of interaction between the different components of social support. The emotional component of social support seems to have no or only a limited contribution to this effect. To describe the complex interactions between social support and cognition and to plan well-directed intervention studies, more detailed research is needed. Future studies should seek to determine which components of social support have a relevant influence on cognition in old age. To achieve this, long observation periods are necessary and standardised operationalisations should be applied.

## Abbreviations

AgeCoDe: German study on ageing, cognition and dementia in primary care patients; FSozU-K14: Questionnaire for social support, 14-item short version; FU2: Follow-up 2; FU3: Follow-up 3; MMST: Mini mental status test; SIDAM: Neuropsychological test battery of the structured interview for the diagnosis of dementia; SISCO: Score of the neuropsychological test battery of the structured interview for the diagnosis of dementia.

## Competing interests

The authors declare that they have no competing interests.

## Authors' contributions

ME has made substantial contributions to conception and design, analysed and interpreted the data and drafted the manuscript. BW has made substantial contributions to conception and design, contributed to the analyses and data interpretation and critically revised the manuscript. SW, SRH, HL, HHK, and WM have made substantial contributions to conception and design and critically revised the manuscript. TZ, MK, KH, FT, DW, JO, MP, AF, JW and ML carried out patient assessments and critically revised the manuscript. MS was involved in data interpretation and the process of drafting the manuscript and critically revised the manuscript. All authors read and approved the final manuscript.

## Pre-publication history

The pre-publication history for this paper can be accessed here:

http://www.biomedcentral.com/1471-2318/12/9/prepub
